# Assessing hemodynamic responses in patients undergoing oral surgery with vasoconstrictor-enhanced local anesthesia: a prospective comparative study between medically compromised and healthy participants

**DOI:** 10.1016/j.clinsp.2026.101222

**Published:** 2026-10-28

**Authors:** Yifat Manor, Fatma Rayyan, Gabriel Gal

**Affiliations:** a Department of Oral and Maxillofacial Surgery the Maurice and Gabriela Goldschleger School of Dental Medicine, Gray Faculty of Medical and Health Sciences, Tel-Aviv University, Tel-Aviv, Israel; b Dental division Oral and Maxillofacial Unit, Shamir Medical Center, Beer Yaacov, Israel; c Tel-Aviv University, Tel-Aviv, Israel

**Keywords:** Hemodynamic responses, Local anesthesia, Oral surgery

## Abstract

**Objectives:**

The present study deals with hemodynamic responses between different types of patients along different stages of operations.

**Methods:**

In a prospective clinical trial,(approved by the IRB approval number 275–15-ASF) and patient informed consent for the procedure, hemodynamic responses were evaluated before and during minor oral surgical procedures performed under local anesthesia. The study encompassed both medically compromised patients (experimental group) and healthy individuals (control group). Hemodynamic parameters, including blood pressure, heart rate, and oxygenation, were monitored. Statistical analysis was conducted to compare parameter fluctuations within each group and patient. Additionally, clinical indicators were documented using a visual analogue scale. Table 1 presents the characteristics of each group regarding age,gender, medical situation and sort of surgical procedure.

**Results:**

The study findings revealed consistent hemodynamic stability across all stages of the procedures. Patients in both groups maintained stable blood pressure, heart rate, and oxygenation levels prior to and during all stages of surgery. Medically compromised patients showed hemodynamic trends comparable to healthy controls. Local anesthesia with vasoconstrictors demonstrated stable profiles and pain management was effective.

**Conclusion:**

Local anesthesia containing adrenaline (1:100,000) demonstrated safety in hemodynamic parameters of healthy and medically compromised patients undergoing minor oral surgery. Overall, hemodynamic fluctuations were minimal, underscoring both the efficacy and safety of using vasoconstrictor-containing local anesthetics for pain management in a controlled hypertension and other well-managed chronic conditions.

## Highlights

• Hemodynamic parameters remain stable across all stages of surgery.
• Adrenaline (1:100,000) provided analgesia without cardiovascular changes.
• Medically compromised patients showed comparable responses to healthy ones.

## Introduction

Local anesthesia plays a pivotal role in modern dentistry, ensuring patient comfort during various dental procedures, especially surgical interventions. One of the crucial effects of local anesthetics is their impact on local vasodilatation. To enhance the precision and control of local anesthesia, vasoconstrictors, such as adrenaline, are often added to the anesthetic solution. This addition not only reduces the distribution of the anesthetic agent but also effectively curtails bleeding during surgical procedures. Furthermore, the inclusion of vasoconstrictors extends the duration of anesthesia and pain control, contributing to a more streamlined and effective patient experience.

Adrenaline, commonly added to lidocaine at a concentration of 1:100,000, introduces a range of benefits to local anesthesia. However, it is imperative to acknowledge potential side effects associated with adrenaline administration. These include elevated systolic blood pressure, reduced diastolic blood pressure, heightened heart rate, and bronchiole dilatation in the lungs.^1^ While local anesthesia without vasoconstrictors may prove less efficacious, it is essential to consider the systemic implications of excessive adrenaline secretion during treatment.^2^ This often prompts dental practitioners to exercise caution when administering local anesthesia containing adrenaline, particularly in patients with compromised medical histories, where vasopressors might be avoided.

The dilemma arises when the desired goals of pain control and minimized bleeding are compromised by concerns over adverse hemodynamic effects. Medically compromised patients frequently necessitate a delicate balance between adequate pain management and avoiding potential complications.

While past studies have investigated hemodynamic fluctuations associated with local anesthesia containing adrenaline in various concentrations,^3^, ^4^, ^5^, ^6^, ^7^ the focus has primarily been on general physiological responses rather than specific oral surgery procedures. The novelty of the present study in comparison to that of Nakamura et al.^4^ and Salma et al.^5^ is that it offers a more granular understanding of how the body physiologically adapts across stages of the procedure. Also, the present study is one of the first to correlate hemodynamic responses with variables such as: anxiety levels or pain scores, duration and type of the dental procedure, use of specific local anesthetics with vasoconstrictors. Also, the study provides evidence-based thresholds or alerts for when hemodynamic changes may indicate a need for clinical intervention or procedural modification, thereby contributing to patient safety protocols during dental surgery ‒ an area which is less developed in earlier works.

This is particularly significant as oral surgery inherently introduces stress for patients due to the nature of the intervention. Therefore, the primary objectives of the present study were to assess whether patients experience hemodynamic fluctuations during minor oral surgery procedures under local anesthesia containing adrenaline and to compare these findings between patients with complex medical histories and those in good health. By scrutinizing these parameters, we aim to shed light on the potential impacts of vasoconstrictors in local anesthesia and provide insights for informed clinical decision-making in oral surgery practice.

## Materials and methods

### Study design

This prospective clinical trial was conducted at “Shamir” medical center and adhered to ethical principles outlined in the Declaration of Helsinki concerning human subject research. The study protocol received approval from the institute's Helsinki committee (275–15-ASF). All participants gave their informed consent to the surgical procedure and for participation in the study. Ethical approval date was initially granted in 31.12.2015 and was subsequently extended throughout the study period until its completion.

### Participants

Participants were recruited between 1.11.2016 to 31.12.2017. Participants were allocated to intervention and control groups according to their systemic status and clinical indication for the procedure (Table 1).

**Table 1:** Demographic and clinical characteristics of participants.

Character	Study group	Control group
Age	63 (35‒96)	37 (35‒79)
Gender	Males ‒ 25	Males ‒ 30
	Females ‒ 44	Females ‒ 33
Health status	ASA 2 ‒ 54	ASA 1 ‒ 63
	ASA 3 ‒ 15	
Surgical procedure	Tooth extraction ‒ 19 patients	Tooth extraction: 44 patients
	Dental implantation ‒ 31 patients	Dental implants ‒ 10 patients
	Biopsy implant exposure and abscess drainage: 9 patients	Biopsy and abscess drainage: 9 patients
Specific medical condition	Cardiovascular disease: 30 patients	Healthy: 63 patients
	Hypertension:13 patients	
	Diabetes: 4	
	Hypothyroidism: 2	
	Combinations: 20	

Patients were categorized based on their American Society of Anesthesiologists (ASA) status,^8^ which denotes their medical fitness. The experimental group comprised medically compromised patients (ASA II and ASA III), while the control group included healthy individuals (ASA I). In medically compromised patients, the use of no more than two carpules of lidocaine 2% with adrenaline 1:100,000 was adopted to minimize cardiovascular risk. This approach aligns with recommendations limiting epinephrine exposure to ≤ 0.04 mg per appointment, a threshold considered safe for patients with cardiovascular comorbidities.^9^^,^^10^ This was also according to the institutional Helsinki committee limitations on this study, up to 2 carpules of local anesthesia containing adrenaline could be used in the study group -medically compromised patients. Inclusion criteria required participants to be aged over 35-years and scheduled for minor oral surgery under local anesthesia with a maximum of 3.6 mL (2 carpules) of lidocaine with adrenaline at a concentration of 1:100,000. For patients in whom adrenaline-containing anesthesia was contraindicated, underwent surgery under plain 3% mepivacaine. Demographic and medical data were collected, and participants were categorized into groups by an oral and maxillofacial surgeon, considering their health complexity as per the ASA classification. Additionally, intraoperative clinical observations, including signs of pain and stress, were meticulously documented. The collected data were summarized in an Excel table for subsequent analysis using SPSS (Statistical Package for the Social Sciences) software.

### Measurements

Throughout the study, participants were subject to continuous monitoring of key hemodynamic parameters, including Systolic Blood Pressure (SBP), Diastolic Blood Pressure (DBP), Heart Rate (HR), and Oxygen Saturation levels (SpO_2_). Measurements were recorded at specific 5 time points: 5-minutes prior to local anesthesia administration (T0), 5-minutes following local anesthesia administration (T1), at the beginning of surgery (T2), at the end of surgery (T3), and 20-minutes after surgery completion (T4). The measurements were performed with noninvasive devices: Microlife© blood pressure device BP 3AG1, for blood pressure and heart rate and for level of oxygenation CONTEC CMS50DL finger pulse oximeter led display. The devices were calibrated monthly in accordance with the manufacturer’s guidelines. Accuracy was verified against a manual mercury sphygmomanometer; cuffs were selected based on arm circumference and placed at heart level while the patient remained seated in a relaxed posture. The CONTEC CMS50DL pulse oximeter was cross-checked weekly using control measurements on healthy individuals.

#### The study diagram

**Patients**
**Healthy /systemic**
**(oral surgery procedures: oral mucosa and gingiva: mucosa biopsy, dental implant exposure, dental abscess drainage.**
**oral mucosa, gums and alveolar bone: tooth extraction, dental implant insertion)**
T0-Monitoring heart rate, blood pressure and oxygen saturation- 5 min before injection
T1-Monitoring heart rate, blood pressure and oxygen saturation- 5 min after local anesthesia injection
T2-Monitoring heart rate, blood pressure and oxygen saturation- at the beginning of surgery
T3-Monitoring heart rate, blood pressure and oxygen saturation- at the end of surgery
T4-Monitoring heart rate, blood pressure and oxygen saturation- 20 min after surgery completion

### Sample size calculation

Sample size was determined based on previous studies that evaluated changes in hemodynamic parameters systolic blood pressure diastolic blood pressure and heart rate (SBP, DBP, HR respectively) during minor oral surgery. Assuming a moderate effect size (Cohen’s *d* = 0.5), a power of 80%, and a significance level (α) of 0.05, a minimum of 34 participants per group was required to detect statistically significant differences in hemodynamic responses across time points. Although the calculated minimum sample size was 68 participants, we included 132 participants to enhance statistical robustness and reliability of subgroup and longitudinal analyses.

### Blinding procedures

Due to the nature of the intervention, the operator was aware of the type of local anesthetic administered. However, hemodynamic parameters were recorded using automated monitoring devices, reducing the potential for measurement bias. To minimize bias hemodynamic data were recorded by a trained assistant not involved in group allocation or outcome interpretation. The data was collected and summarized in an Excel table and the data analyst was blinded to group identity by using coded identifiers. Patients were not informed of specific group assignments to reduce performance bias, although full procedural consent was obtained.

### Statistical analysis

Data were analyzed using IBM SPSS Statistics for Windows, Version 23.0. Descriptive statistics were used to summarize patient demographics and baseline characteristics. To assess changes in hemodynamic parameters across multiple time points, a repeated measures analysis of variance (ANOVA) was performed.

In addition to p-values, effect sizes were calculated using partial eta squared (η²) to determine the magnitude of observed differences both within and between groups over time. Effect sizes were interpreted according to Cohen’s benchmarks. This dual approach ‒ considering both statistical significance and effect magnitude ‒ provided a comprehensive evaluation of hemodynamic responses and helped contextualize the clinical relevance of the findings.

This study adhered to the STROBE (Strengthening the Reporting of Observational Studies in Epidemiology) guidelines, ensuring transparent and comprehensive reporting of observational methodology, participant selection, variable measurement, and statistical analysis.

## Results

### Patient characteristics

Although the calculated minimum sample size was 68 participants, we included 132 participants to enhance statistical robustness and reliability of subgroup and longitudinal analyses. The study cohort consisted of 132 patients, divided into an experimental group and a control group. The experimental group encompassed 69 patients, with an average age of 63-years. 52 patients were operated under Lidocaine 2% with Adrenaline 1:100,000 and 17 patients were treated under Mepivacaine 3%. Among the experimental group, 54 were classified as ASA II and 15 as ASA III. The experimental group exhibited a prevalence of cardiac and blood vessel diseases 43 patients), hypothyroidism (2 patients), diabetes (4 patients), and various combinations of diseases (20 patients). In contrast, the control group included 63 patients with an average age of 37-years, all categorized as ASA I.

### Sample size

The total sample size of 132 patients was based on the expected availability of eligible patients over the study period, while ensuring adequate representation of both medically compromised and healthy individuals. This cohort size exceeds the minimum required for detecting clinically meaningful differences in hemodynamic parameters (e.g., systolic blood pressure or heart rate) between groups.

Table 2 provides data regarding hemodynamic parameters in both groups during the five time points.

**Table 2:** Hemodynamic parameters in both groups along the surgery (during the five time points).

Partial η²	T4	T3	T2	T1	T0	Group	Parameter
0.045	120 ± 21	122 ± 21	128 ± 19	124 ± 17	122 ± 16	Control	SBP (mmHg)
	135 ± 18	138 ± 21	144 ± 22^a^	141 ± 19^a^	140 ± 17	Experimental	
0.030	77 ± 10	78 ± 12	78 ± 13	75 ± 11	76 ± 11	Control	DBP (mmHg)
	78 ± 12	78 ± 12	80 ± 12	78 ± 12	79 ± 12	Experimental	
0.009	71 ± 14	71 ± 13	80 ± 17	77 ± 15	75 ± 15	Control	HR (beat/min)
	75 ± 17	76 ± 17	80 ± 17	78 ± 18	90 ± 10	Experimental	
0.042	98 ± 1.61	98 ± 1.19	98 ± 1.41	98 ± 1.81	98 ± 1.51	Control	SpO_2_ %
	97 ± 1.14	97 ± 1.37	97 ± 1.51	98 ± 1.39	97 ± 1.09	Experimental	

T0 – 5-minutes before local anesthesia administration. T1 – 5-minutes after local anesthesia administration. T2 ‒ At the beginning of the operation. T3 ‒ At the end of the surgery. T4 – 20-minutes after the end of the operation.

Results are given as mean ± standard deviation.

a p 〈 0.00001, ANOVA The clinical thresholds for concern were SBP 〉 180 mmHg, DBP > 110 mmHg, HR > 120 bpm ‒ no patient crossed these thresholds. The clinical thresholds of the hemodynamic parameters were based on institutional protocol according to the recommendations of the ministry of Health in our country.

### Oral surgery procedures

The oral surgery procedures performed on the patients targeted the oral mucosa and gingiva, involving tasks like oral mucosa biopsy, dental implant exposure, dental abscess drainage, tooth extraction (simple or complicated), and dental implant insertion. Notably, the experimental group's procedures predominantly engaged the alveolar bone (72% for alveolar bone, 28% for oral mucosa), while the control group's procedures involved both the alveolar bone (85%) and the oral mucosa (15%) (Fig. 1).

**Fig. 1:**
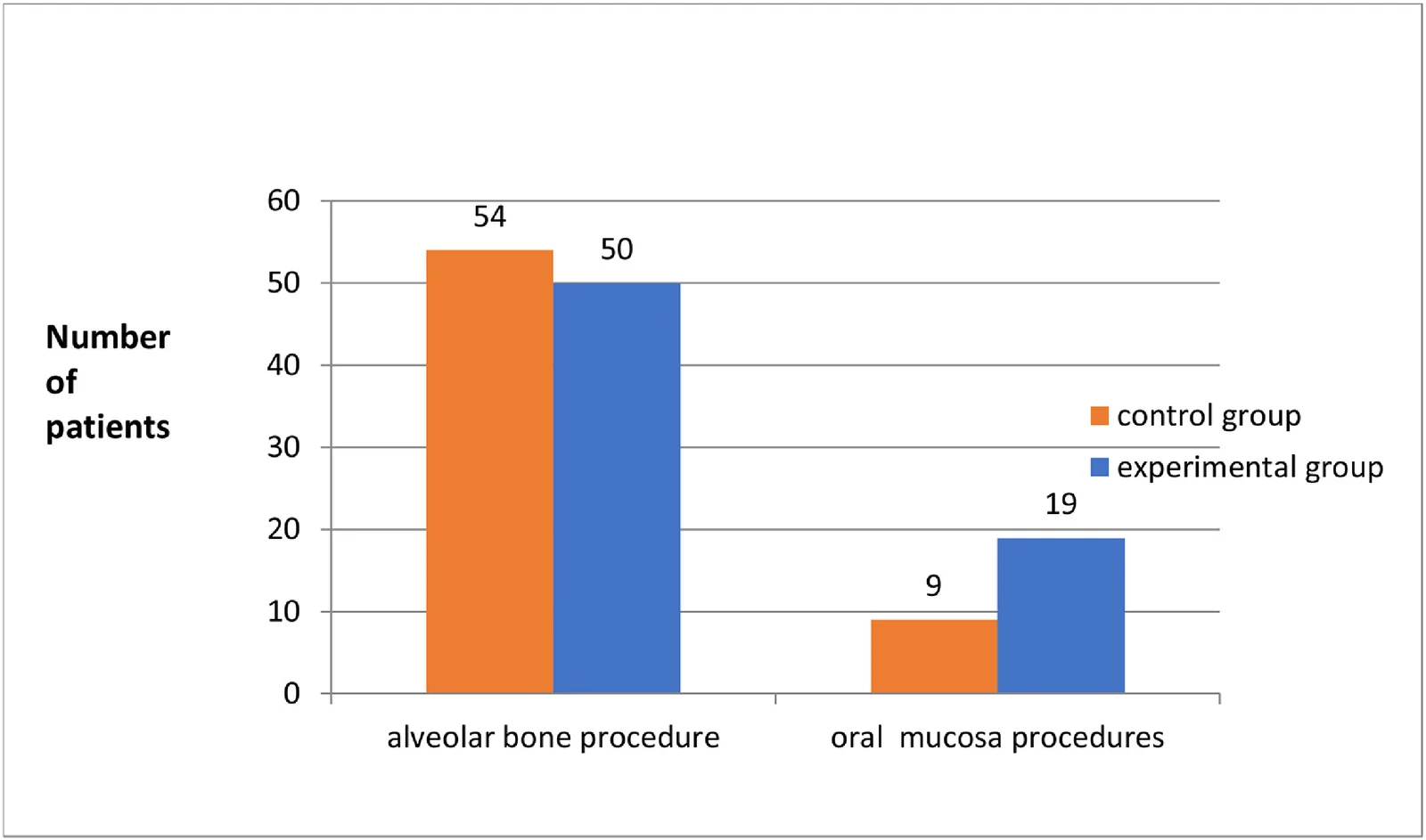
Oral surgery procedures in the study groups.

### Hemodynamic changes

Detailed hemodynamic changes can be found in Table 2, with visual representations of SBP, DBP, HR, and SpO_2_ fluctuations in Fig. 2. Generally, both groups exhibited similar patterns, peaking at T2 at the beginning of the surgery before gradually decreasing. Although the baseline SBP difference between groups was statistically significant (140 vs. 122 mmHg), this finding reflects the expected physiological variation between medically compromised and healthy individuals rather than an anesthetic-related effect. A SBP of 140 mmHg corresponds to stage 2 hypertension according to the 2017 ACC/AHA hypertension guidelines. In addition, no excessive intraoperative SBP elevations (> 180 mmHg), hypertensive crises, or cardiovascular adverse events were observed.

**Fig. 2:**
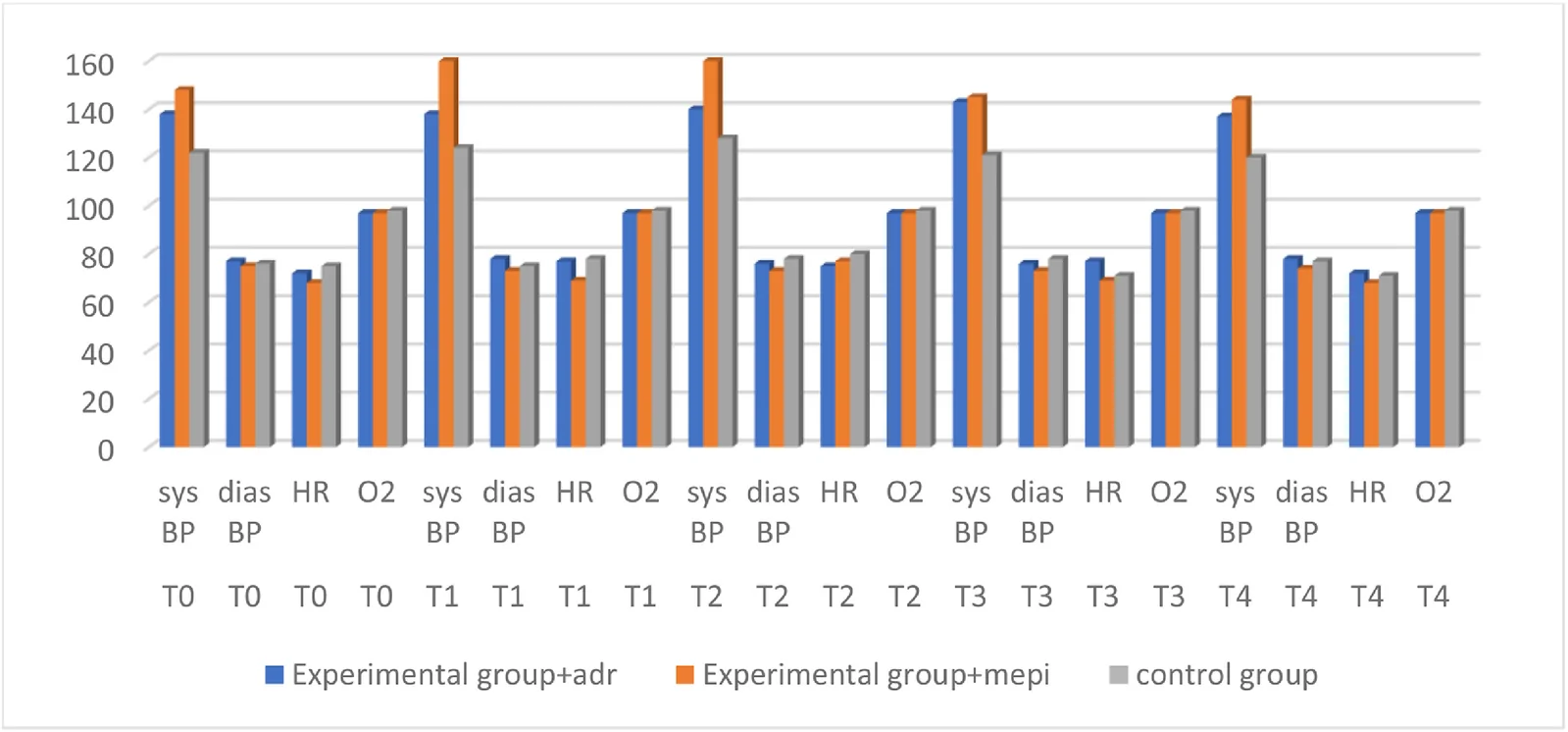
Hemodynamic fluctuations in the study and in the control group. Sys BP, Systolic Blood Pressure; Dias BP, Diastolic Blood Pressure; HR, Heart Rate; O_2_, Level of oxygenation. T0 – 5-minutes before local anesthesia administration. T1 – 5-minutes after local anesthesia administration. T2 ‒ At the beginning of the operation. T3 ‒ At the end of the surgery. T4 – 20-minutes after the end of the operation.

### Systolic blood pressure (SBP)

An ANOVA analysis revealed that changes in Systolic Blood Pressure (SBP) within the same group over time were statistically significant (p < 0.00001). When comparing SBP values between the two groups, a significant difference was found (p < 0.00001); however, the magnitude of the change in SBP did not differ significantly between the groups ‒ meaning the same pattern of change occurred in both. Furthermore, no interaction was observed between the SBP variable and group assignment; in other words, the gap between the groups remained constant across all measurement time points for systolic blood pressure.

Pairwise comparisons with Benjamini-Hochberg correction demonstrated that the expected value of the index at T2 was significantly higher than the corresponding expected values at T0, T3, and T4 (p < 0.05 for all comparisons).

The results should be understood as physiologically expected but not pathologically alarming, reaffirming the safety of the procedures performed under monitored conditions.

### Diastolic blood pressure (DBP), heart rate fluctuations (HR), level of oxygenation (SpO_2_) (Fig. 2)

Throughout the procedures, DBP showed similar trends in both groups with no significant differences at any point. Changes in DBP within and between groups were also non-significant. In contrast to the systolic blood pressure, differences in diastolic blood pressure (η² = 0.030), heart rate (η² = 0.009), and oxygen saturation (η² = 0.042) showed small or negligible effect sizes**,** suggesting minimal practical impact despite s**t**atistical significance in some cases. These differences, although statistically significant, did not exceed clinically accepted thresholds for hemodynamic instability.

Small difference was found in SpO_2_ between the groups (97%vs. 98%). Although it was statistically significant due to the sample size it was within normal physiological limits and clinically irrelevant.

### Correlation between hemodynamics and demographics/clinical parameters

Upon analyzing hemodynamic fluctuations in the experimental group based on age, sex, procedure duration, and pain/stress levels, no correlations were found between hemodynamics and these parameters. The procedure type has no effect on the hemodynamic fluctuation on both groups and was not associated with different hemodynamic response. Additionally, no significant group differences existed in these aspects.

## Discussion

To the best of our knowledge, this is the first study to monitor the effect of local anesthesia containing vasoconstrictors on hemodynamic changes in medically compromised patients during several stages of oral surgery. Medically compromised patients in our country are referred to oral surgery departments in medical centers and are not treated by most oral surgeons in the community. The main concern is the effect of local anesthesia with vasoconstrictors on these patients’ hemodynamic parameters, vital signs, and clinical condition. Most of the physicians prefer to administer local anesthesia without vasoconstrictors, a practice that leads to a relatively short effect of local anesthesia, more bleeding, and sometimes to insufficient levels of anesthesia, resulting to endogenous secretion of adrenaline and causing an increase in HR rate and BP. The purpose of the present study was to determine whether there is a negative effect on hemodynamics in medically compromised patients and to compare their hemodynamics to those of healthy ones. We also compared the clinical conditions of both groups of patients during several stages before and after the administration of local anesthesia and along all the stages of the surgery. The local anesthesia we used was by maximum 3.6 mL (2 carpules) containing 1:100,000 epinephrine. The effect was similar for both the medically compromised and the healthy patients.

### Effect on BP

The averages of the SBP in the study group were higher than in the control group. The mean age of the subjects in the experimental group was higher, so that result could have been expected given that SBP is known to increase with age.^8^, ^11^ Indeed, 55% of the study patients were diagnosed with hypertension, and the “white coat effect” is also more pronounced in the older population.^8^^,^^9^, ^12^ Similar results on hemodynamic fluctuations during oral surgery were found in other studies, with explanations that dental treatment leads to changes in BP in both healthy and hypertensive patients.^4^^,^^5^ Jadhav et al.^6^ recommended that patients with uncontrolled diseases (like diabetes, hypertension) and/or patients having history of cardiovascular complication, should be treated in a medical facility with a readily available cardiology unit. The reason was to facilitates prompt response to emergency treatment in order to reduce morbidity and mortality.

Observing the same trend of fluctuations in SBP and DBP in both groups throughout the monitoring period emphasizes the fact that 3.6 mL of local anesthesia containing vasopressors does not have a different effect on the ASA II and ASA III patients compared to the ASA I patients. The amount of 0.04 mg adrenaline that was injected had minimal effect on hemodynamic parameters of all the patients. The peak values of HR and BP were in the T2 part of the procedure followed by a decrease of the values to values lower than the baseline after it was completed. Nakamura et al.^4^ had described activation of the sympathetic nervous system during dental treatment, leading to a cardiovascular response. This activation began with the administration of anesthesia and peaked during dental surgery; consequently, BP values continued to rise, reaching their maximum during extraction. Ogunlewe et al.^13^ explained the increase in hemodynamic values as being due to physiological and psychological factors, including pain, and Alemany^14^ found the same trend. Jané-Pallí E et al.^15^ claimed that the most stressful part of the procedure is the extraction, and that the values returned to normal 10-minutes after it was completed. Others have argued that just sitting on the dental chair or talking to the dentist can raise the patient’s BP by 5‒6 mmHg, and that the hemodynamic values drop to even lower values than baseline after the procedure when the patient calms down.^16^

In the present study the clinical thresholds for concern was SBP >180 mmHg, DBP > 110 mmHg, HR > 120 bpm. No patient crossed these thresholds.

The changes in hemodynamic parameters ‒ such as systolic and diastolic blood pressure, and heart rate ‒ across procedural time points has no clinical significance. The parameters variations remained within normal physiological limits for the general population and did not necessitate any clinical intervention or affect the overall safety or outcome of the procedure. Similarly, the literature^17^ found that the systolic blood pressure was higher in the hypertensive group before and after the extraction after injection with adrenaline but the changes were not significant.

Sharma et al. observed that internal stress due to fear or pain might cause a release of endogenous adrenaline which can lead to increase in BP.^18^ Those authors found that significant cardiovascular changes occur due to dental anxiety at the time of local anesthesia administration for tooth extraction. Gadve et al.^19^ reported different results: they showed that BP changed significantly during the several stages of the surgery. Their results were related solely to third molar extraction ‒ a procedure that might be more painful and difficult to the patient usually the amount of local anesthetics is higher than in our study. Abu Mostafa et al.^20^ found that the effect of local anesthesia with adrenaline on SBP in hypertensive patients is better than the effect of mepivacaine 3% due to lower pain control. Those authors concluded that it is safe to give controlled hypertensive patients up to 2-carpules of local anesthesia containing 1:80,000 adrenaline, which is a higher concentration than the 1:100,000 we used in our patients.

In the present study, patients receiving local anesthesia with adrenaline demonstrated stable hemodynamic parameters and effective pain control and the results support its safe use in appropriately selected patients. It can be suggested that effective analgesia may attenuate endogenous adrenaline release and thereby blunt sympathetic cardiovascular responses. The literature reports about the physiological effects of pain-related stress and catecholamine release.^21^, ^22^, ^23^ The present study found this effect in both groups.

It can be speculated that higher doses of local anesthesia containing adrenaline, more than the two carpules we used, might cause fluctuations in BP and HR especially in medically compromised patients. Oral surgery under general anesthesia also entails the use of local anesthesia containing adrenaline for local hemostasis and prevention of pain. The amount of injected local anesthesia is usually higher than 2 carpules. It may be interesting to evaluate the hemodynamic parameters under higher amounts of local anesthesia than the ones we used, although general anesthesia itself has an effect on hemodynamic parameters.

It has been suggested that the level of procedural invasiveness may influence hemodynamic changes. Salma et al.^24^ reported that more invasive procedures, such as tooth extraction, are associated with greater increases in blood pressure compared to less invasive procedures like restorations or scaling. However, in the present study, no such effect was observed when comparing procedure type (oral mucosal versus alveolar surgery) across the evaluated parameters.

Another consideration is the potential impact of patient anxiety on hemodynamic parameters. A previous study in healthy adult patients demonstrated that anxiety related to the type of dental procedure may contribute to hemodynamic changes.^24^ Although the present study did not find this effect, close monitoring of patients ‒ particularly those with dental anxiety ‒ is recommended during treatment both in healthy and medically compromised individuals.

### Severity of systemic disease

The severity of several medical problems of the experimental group did not affect the hemodynamic changes, and no difference was found between the experimental and control groups. Niwa et al.,^7^ examined hemodynamic parameters under local anesthesia without surgical intervention among subjects with cardiovascular disease according to ASA status I-III and observed no difference in hemodynamic changes among the subjects in the different groups. The present study analyzed the effect of local anesthesia and surgical intervention, found that the severity of the medical status did not affect the hemodynamic changes.

### Effect on heart rate (HR)

Same fluctuations in HR were found in both groups. There was a rise in HR from the initiation until the middle of the procedure followed by a decrease to baseline levels. The similarity in HR dynamics for both groups was most probably as a result of the medical treatment of the experimental group. The adrenaline and the stress of the oral surgery procedure did not cause a rise or fluctuation in HR.

### Oxygen saturation

The small difference in SpO_2_ between groups (97%vs. 98%) was statistically significant due to the large sample size but is within normal physiological limits and clinically irrelevant. The SpO_2_ level did not change throughout the procedure in both groups and no cyanosis or other clinical signs of oxygenation problems were found.

### Limitations of the study

This study has several limitations. First, as a non-randomized prospective cohort design, the possibility of residual confounding and selection bias cannot be entirely excluded. Second, operator blinding to the type of local anesthetic was not feasible due to the nature of the intervention, although outcome measurements were obtained using objective automated monitoring devices.

### Future directions

This is a pilot study to find out the trends of hemodynamics in oral surgery patients. Future studies will analyze the fluctuation by a 24-hour monitoring machine ‒ Holter for each oral surgery procedure and for each cardiology diagnosis and will also look for the relationship between the effect of psychological situation on the hemodynamics.

## Conclusions

In light of the limitations inherent to the present study, several key conclusions can be drawn. Our investigation has demonstrated that minor oral surgery, involving the administration of up to 3.6 mL of local anesthesia comprising lidocaine and adrenaline at a concentration of 1:100,000, does not result in substantial hemodynamic effects. This practice proves to be safe, even for patients with stable controlled hypertension and other cardiovascular conditions. Furthermore, the hemodynamic responses observed in those patients were analogous to those witnessed in the healthy control group. Notably, neither systemic disease nor surgical procedures yielded any complications.

Local anesthesia containing adrenaline (1:100,000) was associated with effective pain control and stable hemodynamic parameters during minor oral surgery in the observed cohort. Further trials are needed to evaluate the hemodynamic impact of different local anesthetics in diverse patient populations.

These conclusions collectively underscore the feasibility and safety of employing local anesthesia with vasoconstrictors for pain control during minor oral surgery, particularly in patients presenting with stable controlled hypertension and other well-managed chronic conditions. As future research may delve into higher doses of local anesthesia and the potential interactions with general anesthesia, our study offers a pivotal foundation, promoting both the effectiveness and security of this approach.

## Declarations

The authors used AI assisted language edition by Chat GPT. The author reviewed and edited the content as needed and take full responsibility for the content of the publication.

## Author’s contribution

Yifat Manor: Conceptualization; methodology; visualization; project administrator drafting; writing-review and editing; final approval.

Fatma Rayyan: Data curation; visualization; drafting and final approval.

Gabriel Gal: Supervision; data analysis; drafting and final approval.

All authors agree to be accountable for all aspects of the work in ensuring that questions related to the accuracy or integrity of any part of the work are appropriately investigated and resolved.

## Declaration of competing interest

The authors declare no conflicts of interest.

## Data Availability

Most of the data are available within the text, and the missing data will be available from the corresponding author on reasonable request.
